# Suicide victims and alcohol-related consumption in Brazil: An observational study and a narrative review of the literature

**DOI:** 10.1007/s12024-023-00766-4

**Published:** 2023-12-27

**Authors:** Ivan Dieb Miziara, Carmen Silvia Molleis Galego Miziara

**Affiliations:** 1 Legal Medicine, Sao Paulo University Faculty of Medicine, Rua Teodoro Sampaio, São Paulo, SP 352-22 Brazil; 2 Legal Medicine, ABC Faculty of Medicine, Santo André, Brazil

**Keywords:** Suicide, Alcohol, Hanging, Firearm

## Abstract

Deaths due to external causes, mainly suicide, are a severe public health problem in Brazil. Evidence shows that the tendency to impulsive behavior is exacerbated after alcohol consumption. The relationship between alcohol and suicide is poorly described in the medical literature. The study aims to analyze the relationship between victims' blood alcohol levels and suicides in some municipalities in Greater São Paulo, Brazil. We reviewed the data from the medical records of 805 necropsies performed at the Medical Legal Institute of Sao Paulo in Franco da Rocha, Brazil, from 2001 to 2017. The manner of death was established based on the result of police inquiry. Deaths due to suicide (n=41) were selected for the study. Descriptive statistics and Student t-test was applied when appropriate. The variables studied were sex, age, suicide mechanism, and blood alcohol level (BAC). In all cases we could not determine how much time the deceased consumed alcohol before suicide. Of the individuals analyzed, 85.36% were male, and 14.64% were female. The most prevalent age range for males was between 18 and 23 (19.5%). For females, it was between 12 and 23 years (33.2%). Most suicides (48.78%) were due to hanging, followed by self-poisoning (22.08%) and firearms (17.1%). 38 victims (92.68%) presented a positive BAC, over 0.3 mg/dl. The higher levels were in the group of suicide by hanging (2.3 mg/ml). Thus, alcohol intoxication is common among suicide victims, and it can contribute to the fatal outcome as a risk factor. Further studies are necessary for a better comprehension of the effect of alcohol on suicide victims.

## Introduction

### Suicide definition and risk factors

Suicide is an external cause of death that has intrigued man and science for centuries. Suicide is a complex phenomenon resulting from the interaction of several factors. Much has been speculated about the factors that lead an individual to commit intentional suicide. In general, some risk factors are associated with the execution of the suicidal act—for example, mental disorders and alcohol consumption.

More recently, researchers have postulated the existence of biomarkers as risk factors for suicide. As stated by Capuzzi et al., “suicidal risk may be increased by abnormalities in the serotonergic system, the hypothalamic‒pituitary‒adrenal (HPA) axis, lipid metabolism, the immune system, and neuronal plasticity” [1, 2].

Braquehais et al. [2] stated that “an abnormal HPA axis functioning together with an anomalous interaction between HPA mechanisms and other systems such as the serotonergic system may be one of the neurobiological correlates of emotional dysregulation.” In their review, the authors hypothesized that this dysregulation “may play an important role in adolescent suicidal behavior” [2]. Berardelli et al. found that “HPA axis activity is involved in suicide risk, regardless of the presence or absence of psychiatric conditions,” and they proposed that “targeting HPA axis dysregulation might represent a fruitful strategy for identifying new treatment targets and improving suicide risk prediction" [3].

However, some authors point out that other factors may play a role in suicidal ideation or suicidal behavior, such as demoralization. Costanza et al. pointed out that the association between demoralization and suicidal ideation or suicidal behavior “may be trans-nosographic” and “may contribute to more comprehensive suicide risk detection” [4].

On the other hand, some authors point out that peripheral biomarkers can help in the development of suicide prevention strategies. These peripheral biomarkers are easily accessible, cost-effective, and minimally invasive. The authors found that individuals who had recently attempted suicide had low levels of interleukin-2, and patients at risk for suicide had high levels of interleukin-6 in their cerebrospinal fluid [5].

Other authors, such as Xu et al., demonstrated that cytokine levels in peripheral blood are potential biomarkers of suicidal ideation in patients with major depressive disorder. A study of 55 patients found higher levels of interleukin-17C (IL-17C), C-X-C motif chemokine ligand 10 (CXCL10), and tumor necrosis factor-beta (TNF-B) in individuals with suicidal ideation [6].

### Alcohol and suicide

Suicide is recognized as one of the leading external causes of death, accounting for approximately 800,000 deaths per year worldwide [7, 8]. The acute or chronic use of alcohol is known to be a factor capable of altering individuals' behavior, resulting in a tendency to commit suicide in many cases [8]. A study carried out in New Zealand recently demonstrated that the association of alcohol dependence with suicidal ideation was significant [9]. Suicide types include simple, complex, and complicated suicide [10]. Suicide using a single method is defined as simple, but suicide using two or more methods is defined as complex or complicated. “Complex suicide” refers to a form of suicide in which two or more methods are applied simultaneously or in chronological succession, and “complicated suicide” involves suicide attempts in which the chosen method fails. “Complicated suicide” involves another type of traumatization [10]; however, it is a rare type of suicide that can be very difficult to investigate and determine the cause of death as either suicidal or accidental.

It is also well known that alcohol may trigger self-destructive behavior, leading to depressive thoughts, impaired coping strategies to avoid psychological distress, and so on [11]. In addition, alcohol abuse is a “significant global public health problem, accounting for 1.3% of all deaths, which is 700,000 lives each year” [12]. Cherpitel et al. reviewed studies of acute alcohol use and suicidal behavior published over ten years (1991–2001) and found a wide range of alcohol-positive cases involving both suicide (10–69%) and suicide attempts (10–73%) [13]. In a retrospective psychological autopsy study, Kolves et al. reported that 68% of males and 29% of females who committed suicide met the criteria for alcohol abuse or dependence [14]. Thus, there is variable support for a direct link between alcohol consumption and suicide from some medical literature data from different countries. Longitudinal and cross-sectional aggregate-level studies have reported a significant and positive association between alcohol consumption and suicide [13–18].

Deaths due to external causes are a severe public health problem in Brazil—the third leading cause of mortality in the country [19]; the leading cause of death is cardiovascular disease, and the second leading cause is malignant neoplasms. In addition, suicide is one of the most important of these external causes in Brazil, along with homicides and traffic accidents. Most authors agree that the role of alcohol is crucial in understanding this phenomenon [20, 21]. There is also evidence that the tendency toward impulsive and violent behavior is exacerbated after alcohol consumption [22].

Nevertheless, the relationship between alcohol consumption and violent deaths, mainly suicides, is described in the medical literature but lacks precision. For this reason, some authors believe that “alcohol as a relevant factor in cases of sudden, unexpected or unnatural death seems to be underestimated” [20].

It is also important to emphasize that alcohol consumption in Brazil is characteristically high. A national household survey estimated a prevalence of 74.6% for alcohol use (socially) and 12.3% for alcohol dependence in the studied population aged 12 to 65 years [21].

For 632 cases of suicide in the city of São Paulo in 2005, Ponce et al. [22] concluded that 33.1% of the victims had high blood alcohol levels, with a higher prevalence among males than females (37.1% versus 20.1%, respectively). They concluded that one-third of the sample committed suicide after alcohol consumption. In a study published in 2018 in São Paulo, alcohol was the primary substance consumed before the fatal event [23].

### Objectives and hypothesis of the study

As we mentioned, the correlation between alcohol use and abuse and suicide deaths among fatal victims is still poorly studied in Brazil. Thus, the main objective of the present study was to address this deficit using Medical Legal Institute of the State of Sao Paulo (IML-SP) autopsy data on suicide and alcohol poisoning deaths from 2001 to 2017. We also aimed to analyze the relationship between blood alcohol levels and suicide deaths in the microregion formed by some municipalities in Greater São Paulo (Franco da Rocha, Caieiras, Mairiporã, and Francisco Morato), which is served by the Medical Expertise Team Franco da Rocha of the Legal Medical Institute of the State of São Paulo. These municipalities are characterized as suburbs of the city and serve only as a “dormitory” for the population that works in São Paulo.

Furthermore, the hypothesis of the study was to demonstrate the correlation between alcohol use and suicide cases in Brazil. Additionally, we attempted to show that alcohol consumption is a significant risk factor for suicide, and it should be considered in the development of suicide prevention policies.

It is also important to note that, under the law in Brazil, all cases of death of nonnatural origin, that is, suicide deaths and cases of suspected violence, must be submitted for autopsy at the Medical Legal Institute to verify the exact nature of death.

## Methods

The study was approved by the Ethics Committee for Analysis of Research Projects (CAPPesq) of the Faculty of Medicine of the University of São Paulo under number 0090/09 on 03/11/2019 and by the Scientific Committee of the Medical Legal Institute of the State of São Paulo.

In Brazil, suicide cases are considered violent deaths and investigated as criminal cases by the prosecutor's office. Therefore, the primary outcome measure was the presence of alcohol in the blood of deceased suicide victims.

Retrospectively, we reviewed the data from the medical records for 805 necropsies performed at IML-SP in Franco da Rocha, Brazil, from 2001 to 2017. We excluded 764 cases for which the autopsy indicated death from natural causes (n = 158) or other violent causes (n = 606) from the analysis. We included only 41 cases of suicide in the study.

We statistically analyzed the data obtained using the SPS program. In addition, we calculated descriptive statistics to assess the overall blood alcohol concentration (BAC) of the decedents. The variables studied were sex, age, manner of death, and BAC. Mean comparisons were performed with Student's *t* test when the groups showed normal distributions. Otherwise, nonparametric Wilcoxon or Mann‒Whitney U tests were used. P < 0.05 was considered significant.

We considered a BAC above 0.3 mg/dl to be high (the limit Brazilian law imposes on drivers when driving a vehicle).

We collected 10-ml blood samples during autopsies, preferably taken from the right femoral vein, to perform the BAC measurement. The center of Forensic Toxicology of the IML determined the alcohol concentration by gas chromatography using the “head space” and double column separation techniques.

## Results

The demographic characteristics of the group including all individuals with violent deaths are shown in Table 1. Of the 647 individuals analyzed, who died a violent death, 547 (84.5%) were male, and 100 (15.5%) were female. The individuals were divided into different age groups to facilitate analysis. The distribution of the age groups according to sex is also shown in Table 1. The average age for males was 30 years (21–43), and for females, it was 26 years.

**Table 1:** Age of the 617 individuals whose autopsies indicated death due to violent causes according to sex (2001–2017)

	Sex			
	Male	Female	P	Total
	(n = 532)	(n = 85)		(n = 617)
Age (years)	30 (21–43)	26 (17–49)	0.49	30 (21–44)
0–4	15 (2.8%)	6 (7.1%)	0.02	21 (3.4%)
5–11	7 (1..3%)	5 (5.9%)		12 (1.9%)
12–17	48 (9..0%)	11 (12.9%)		59 (9.6%)
18–23	99 (18..6%)	11 (12.9%)		110 (17.8%)
24–29	94 (17.7%)	12 (12.9%)		105 (17.0%)
30–39	95 (17.9%)	12 (14.1%)		107 (17.3%)
40–49	92 (17.3%)	9 (10.6%)		101 (16.4%)
50–59	47 (8.6%)	10 (11.8%)		57 (9.2%)
60 or more s	35 (6.6%)	10 (11.8%)		45 (7.3%)

Data are presented as the median (p25–p75) and n (%)

Table 2 shows the demographic characteristics of the 41 deceased individuals in the suicide group. There were 35 male individuals (85.36%) and six female individuals (14.64%). The median age range in the male group was 18–23 years (19.5%), and that in the female group was 12–23 years (33.2%). There was a significant difference between these groups (P < 0.05).

**Table 2:** Age range of the 41 suicide victims according to sex (2001–2017)

Sex	Male	Female	p
N	35 (85.36%)	6 (14.64%)	< 0.05
Age range	18–23 (19.5%)	12–23 (33.2%)	< 0.05

Of the total of 647 violent deaths, only 41 (6.33%) were due to suicide. A total of 230 deaths were due to homicide (35.54%), and 181 deaths (27.97%) were due to traffic accidents. We did not identify any deaths of undetermined cause.

The mechanism of death in suicide groups related to all violent deaths is presented in Table 3. Death by hanging was the most frequent type of suicide (n = 20, 48.8%), followed by intoxication (n = 9, 22%). There were 7 cases of suicide by firearm (17.1%), 2 cases of suicide by sharp force trauma (4.9%), and three cases of suicide by jumping from a height (7.2%).

**Table 3:** Mechanism of death in suicide cases related to the total number of violent deaths (2001–2007)

**Cause**	**N**	**% group**	**% Total (617 individuals)**
**Suicide**			
Hanging	20	48.80%	3.20%
Intoxication (self-poisoning)	9	22.00%	1.50%
Firearm	7	17.10%	1.10%
Sharp trauma	2	4.90%	0.30%
Jumping from a height	3	7.20%	0.50%
*Total*	*41*	*100.00%*	*6.60%*

Table 4 presents the prevalence and median BAC for the suicide group. Only 38 suicide victims had available BAC data. The BAC was higher in individuals who died from suicide by hanging (2.3 mg/ml). Importantly, the two individuals who committed suicide by jumping from a height did not have any alcohol in their blood.

**Table 4:** Prevalence and median blood alcohol concentrations in suicide victims (2001–2007)

**Cause**	**N (%)**	**High blood alcohol concentration**	**Blood alcohol concentration (mg/ml)**
	**Available cases**	**(> 0.3 mg/ml) in%**	**(in positive cases)**
**Suicide**			
**Hanging**	19 (95.0%)	7 (36.8%)	2.3 (0.8–2.8)^*^
**Intoxication(self-poisoning)**	8 (88.9%)	0 (0.0%)	-
**Firearm**	7 (100.0%)	1 (14.3%)	1.5^*^
**Sharp force trauma**	2 (100.0%)	1 (50.0%)	1.7^*^
**Jumping from a height**	2 (66.7%)	0 (0.0%)	-
***Total***	38 (92.7%)	9 (23.7%)	2.2 (1.2–2.7)^*^

^*^Values ​​presented as the median (p25–75)

## Discussion

The present study's results are essential for clinical and preventative to understand suicide pathophysiology. The association between alcohol use and suicide is indisputable. In addition, the median BAC in our series was high enough to ensure the association. We did not know the time interval between the consumption of alcohol and the act of suicide; therefore, the concentrations found were high enough for us to assume that the ingested amounts were either very large or were consumed in a time interval very close to the initiation of suicidal action.

We found a high median BAC in individuals who committed suicide by hanging (2.3 mg/ml), by firearm (1.5 mg/ml), and by sharp force trauma (1.7 mg/ml) and a total average of 2.2 mg/ml in all cases. Although additional research in this area is needed. If further results confirm our results, it will be necessary to conduct routine screening for suicidal ideation and behavior in all heavily drinking individuals who present to a healthcare facility (both in primary care and emergency room settings).

The present study also confirms some findings from other researchers on this topic (the association between the BAC and death from unnatural causes) and concerning suicide victims [7, 8, 12, 13, 15]. In this case, concerning the sex of the individuals involved, our study confirms the findings of Ponce et al. [22]. Most of the individuals who committed suicide in our sample were male (85.36%), with statistical significance (p value < 0.05). According to Yeskendir et al. [12], who found that "males used alcohol more commonly before committing suicide than females," our findings still hold true. Moreover, related to the age group, in general, suicidal males were older than suicidal females, with statistical significance (p value < 0.05). In our sample, they were young adult males (18 to 23 years of age).

However, it is essential to highlight the high incidence of the problem among female adolescents (aged 12 to 23 years), corresponding to 33.2% of the individuals studied. Our findings agree with Carlini et al. [19] regarding high alcohol consumption in this age group in Brazil. The predominance of female adolescents in our suicidal sample is intriguing. This finding may indicate behavioral changes in this age group, which deserves more accurate investigation. In one individual in the female suicidal group, death occurred after an episode of rape by her stepfather. According to information from the victim’s relatives, the 14-year-old girl became depressed and was found dead beside an empty whiskey bottle. In this particular case, one might ask whether the death was suicidal or whether it was an unintentional alcohol overdose. However, the BAC of this victim (0.8 mg/ml) did not reach the lethal dose of alcohol, which is usually above 4.0 mg/l [24].

As stated by Perez et al. [25], “two of the most common psychiatric conditions found in suicide decedents are major depressive disorder and substance use disorder… A depressive disorder creates a significant risk for death by suicide” [26–29]. Therefore, we can speculate that female adolescents are more prone to depression (a common problem in this age group), and alcohol consumption becomes a trigger for or precedes a suicide attempt [30, 31]. Lasota et al. [28] reported that “differences in sex proportions are also related to age. There are many reasons why female and male suicide rates differ. These include gender equality, differences in coping with stress and conflict, the availability and preference of different ways of dying by suicide, the availability and patterns of drinking alcohol, and differences in the rates of seeking help for mental disorders by women and men.”

In addition, “problems with depression and alcohol abuse often occur together” [31], and it seems that major depression has a causal influence on subsequent alcohol dependence [32]. It becomes a vicious cycle: “Depressed patients often struggle with harmful alcohol use, and alcohol abuse patients often struggle with depression” [31]. Even when these patients are receiving treatment, the combined presence of depression and alcohol dependence increase their suicidal thoughts and suicide attempts [30–32].

### Peculiar aspects of our series

In our series, some aspects call for attention. First, we noted some association between the alcohol concentration and the mechanism of suicide attempts. The highest concentrations of alcohol in the blood were associated with suicide by hanging. However, we did not determine the BAC for individuals who died from suicide by jumping from a height or suicide by intoxication. Thus, we agree with the statement made by Sher et al. [32] that “when alcohol is used deliberately to impair judgment or lower inhibitions against suicide, the suicidal actions tend to become more lethal.”

Second, the use of firearms and sharp force trauma to commit suicide seems to be associated with a high BAC. In our sample, seven individuals who used a firearm presented a median BAC of 1.5 mg/l. Two victims who used sharp force trauma showed a median BAC of 1.7 mg/l. These data confirm the recent findings by Lange et al. [33], who stated that “the more alcohol consumed, the higher the probability of using one of the most lethal methods of suicide”, including a firearm or knife. Suicide attempts with a firearm result in death in 90% of cases [33].

Third, the region covered by the Medico-Legal Institute in Franco da Rocha is one of the poorest regions of the State of Sao Paulo (Brazil), the country's wealthiest state. This fact confirms that reported by Udesen et al. [34], who stated that “there was substantial socioeconomic inequality in alcohol-related deaths.”

Finally, it is essential to remember that although suicide attempts by self-poisoning accounted for the minority of cases (n = 9, six females, three males), these results align with those found by Salles et al. [35] in a much larger population (516 patients admitted to the emergency department for self-poisoning).

### Questions and possible answers

Relevant questions regarding public health arise in our study: to what extent did the drunk victim contribute to their death? The answer is evident in cases of drowning, falling, car accidents in which the driver was drunk and aspiration of pieces of meat during a barbecue. However, what happens in suicide?

We know that the action of alcohol in the body has two main phases. First, alcohol inhibits inhibitory synapses in the central nervous system. It encourages impulsive, often violent, behavior, which can give rise to an aggressive attitude on the part of the aggressor. In the second phase, alcohol acts as a depressant in the central nervous system, causing an individual to have reduced volition and attention, the ability to resist aggression, and the ability to drive vehicles, which can lead to accidents, such as drowning, falls, and swallowing disorders. However, again, what happens in suicide?

Furthermore, alcohol use can increase the risk for suicide attempts “by heightening psychological distress and impulsivity, while temporarily decreasing cognitive processing and problem-solving” [30]. Although not very well explained, the relationship between alcohol use and suicide is indisputable; in a recent meta-analysis, Isaacs et al. [8] demonstrated a strong connection “between problematic/heavy alcohol use and subsequent death by suicide.”

What’s worse, the effects of alcohol use on suicide outcomes are often long-lasting. Some studies show that adolescents who used alcohol within the past year were at an increased risk of suicidal ideation in that same year [36–38]. On the other hand, Sher et al. [32] postulated that in “individuals with comorbid depression and alcoholism, a greater serotonergic impairment may be associated with a higher risk of committing suicide and that dopaminergic dysfunction may play an important role in the pathophysiology of suicidal behavior in people with alcoholism.”

Additionally, what explains the relationship between high BACs and firearm suicide or suicide due to sharp force trauma? We did not find an explanation in the literature. The answers to these questions are not simple. Perhaps further studies are needed to correlate the crime scene and the genesis of the events to find a satisfactory explanation.

Our study has some limitations, the first being its cross-sectional design, which did not enable us to address causal relationships between the BAC and death by suicide. Due to the nature of our study, we can only highlight the association between alcohol use and suicide. As we said, it is impossible to establish a cause-and-effect relationship under these conditions. Furthermore, we were unable to verify the time interval between the use of alcohol and the suicidal act.

There are limitations concerning BAC measures, as we did not assess the time of the last drink before the suicide occurred. This could lead to a false negative result, suggesting that no alcohol was used (particularly in cases of self-poisoning and jumping from a height).

On the other hand, our study has some strengths: the number of cases was high (compared to other similar studies) and the alcohol/suicide association was unmistakable, and it should be used to alert public health authorities to take measures to prevent the problem.

### Final considerations

The association between alcohol and violence has been understood throughout the ages. Many individuals under the influence of alcohol have the courage to commit violent acts, including against themselves. Our study showed that the victims being drunk contributed significantly to the fatal outcome. This is true not only in other cases of violent deaths (e.g., homicides, traffic accidents) but also in cases of suicide. In the case of accidents, the BAC was a direct contributing factor. In contrast, in other cases, the contribution was made indirectly but was no less critical in the fatal outcome.

Importantly, our study has some limitations, the first being its cross-sectional design, which did not enable us to address causal relationships between our BAC results and death by suicide. There are limitations concerning BAC measurement, as we did not assess the time of the last drink before the suicide occurred. This fact can lead to a false negative suggestion that no alcohol was used (particularly in the cases of self-poisoning and jumping from height).

It is also important to emphasize that our study has some strengths: the number of included cases was high (compared to similar studies). This demonstrated that, in some way, the alcohol/suicide association is unequivocal. Again, this result should be used to alert public health authorities to take measures to prevent the problem. One of these measures is identifying and treating individuals with drinking problems and suicidal ideation, screening these individuals (mostly those with suicidal ideation) using peripheral biomarkers and promoting specific therapeutic options for this group of individuals. As suicide by firearm, falling from a height, and self-poisoning are some of the most lethal forms, other public action measures are needed, such as restrictions to accessing firearms and some pesticides, neurotropic medications, and illicit drugs. These measures must be implemented or reinforced in Brazil. Conversely, public spaces such as viaducts and bridges must be built with strict protection rules.

## Conclusions

As we explained in the Discussion section, some conclusions can be drawn:

1. Our study showed that the fact that the victims were drunk could have contributed significantly to the fatal outcome in suicide cases.
2. Due to the cross-sectional nature of our research, we cannot say that there is a cause‒effect relation.
3. Nevertheless, it seems that there is a clear association of the BAC with suicide, which needs consideration in the explanation of suicide pathophysiology, motivation, or ideation.
4. The BAC an indirect contributing factor. Thus, we can assume it was no less critical in the fatal outcome.
5. Suicide prevention programs need to consider some points, and some measures must be taken, namely, the treatment of alcohol dependence; the prevention of alcohol use by adolescents, especially those with depressive disorders; the restriction of access to firearms; the construction of protective structures in elevated locations, such as bridges, buildings, and viaducts; and the use of peripheral biomarkers as a screening method, especially in individuals with suicidal ideation.

## Key points

1. The association between alcohol and suicide is quite clear
2. Using more effective forms of instruments to cause death (firearm, sharp force trauma) is associated with higher levels of BAC
3. Suicide prevention programs must include treatment of alcohol dependence, restriction of access to firearms, and construction of protective structures in elevated locations

## Data Availability

NA.

## Code availability

NA.

## References

[1] Capuzzi E, Caldiroli A, Capellazzi M, et al. Biomarkers of suicidal Behaviors: A comprehensive critical review. Adv Clin Chem. 2019;96:179–216. 10.1016/bs.acc.2019.11.005. 32362318 10.1016/bs.acc.2019.11.005

[2] Braquehais MD, Picouto MD, Casas M, et al. Hypothlamic-pituitary-adrenal axis dysfunction as a neurobiological correlate of emotional dysregulation in adolescent suicide. World J Pediatr. 2012;8(3):197–206. 22886191 10.1007/s12519-012-0358-0

[3] Berardelli I, Serafini G, Cortese N, et al. The Involvement of Hypothalamus-Pituitary-Adrenal (HPA) Axis in Suicide Risk. Brain Sci. 2020;10:653. 10.3390/brainsci10090653. 10.3390/brainsci10090653 PMC7565104 32967089

[4] Costanza A, Vasileios C, Ambrosetti J, et al. Demoralization in suicide: A systematic review. J Psychosom Res. 2022;157:110788. 10.1016/J.psychores.2022.110788. 35334350 10.1016/j.jpsychores.2022.110788

[5] Kang HJ, Kim JW, Kim SW, et al. Peripheral Markers of Suicidal Behavior: Current Findings and Clinical Implications. Clinical Psychopharmacology and Neuroscience. 2023;21(4):650–64. 10.9758/cpn.22.1046. 37859438 10.9758/cpn.22.1046 PMC10591170

[6] Xu Y, Liang J, Gao W, et al. Peripheral blood cytokines as potential diagnostic biomarkers of suicidal ideation in patients with first-episode drug-naïve major depressive disorder. Front.Public Health. 2022. 10.3389/fpubh.2022.1021309. 10.3389/fpubh.2022.1021309 PMC9678225 36420006

[7] Razvodovsky YE. Alcohol and Suicide in Belarus. Psychiatr Danub. 2009;21(3):290–6. 19794344

[8] Isaacs JY, Smith MM, Sherry SB, et al. Alcohol use and death by suicide: A meta-analysis of 33 studies. Suicide Life Threat Behav. 2022;52:600–14. 35181905 10.1111/sltb.12846

[9] Crossin R, Cleland L, McLeod GFH, et al. The association between alcohol use disorder and suicidal ideation in a New Zealand birth cohort. Aust N Z J Psychiatry. 2022;56(2):1576–86. 10.1177/00048674211064183. 34903072 10.1177/00048674211064183

[10] Beltrame B, Baig S, Verzeletti A. Forensic remarks regarding 35 cases of complex suicides and 4 cases of complicated suicides investigated at the Institute of Legal Medicine of Brescia during the period 1983–2022. Legal Med. 2023;65:102324. 10.1016/j.legalmed.2023.102324. 10.1016/j.legalmed.2023.102324 37738750

[11] Hufford MR. Alcohol and suicidal behavior. Clin Psychol Rev. 2001;21:797–811. 11434231 10.1016/s0272-7358(00)00070-2

[12] Yeskendir A, Elsenberg D, Kaplan MS. Acute use of alcohol before suicide in Kazakhstan: A population-wide study. J Affect Disord. 2023;321:134–9. 36272459 10.1016/j.jad.2022.10.017

[13] Cherpitel CJ, Borges LG, Wilcox HC. Acute alcohol use and suicidal behavior: a review of the literature. Alcoholism. 2004;28:18–28. 10.1097/01.alc.0000127411.61634.14 15166633

[14] Kolves K, Vatnik A, Tooding LM, Wasserman D. The role of alcohol in suicide: a case-control psychological autopsy study. Psychol Med. 2006;2:1–8. 10.1017/S0033291706007707 16650347

[15] Caces P, Harford T. Time series analyses of alcohol consumption and suicide mortality in the US, 1984–1987. J Studies Alcohol. 1998;59:455–61. 10.15288/jsa.1998.59.455 9647428

[16] Marusic A. Suicide in Slovenia: Lessons from cross-cultural psychiatry. Int Rev Psychiatry. 1999;2:213–20.

[17] Razvodovsky YE. The association between the level of alcohol consumption per capita and suicide rate: results of time-series analysis. Alcoholism. 2001;2:35–43.

[18] Pridemore WA, Chamlin MB. A time-series analysis of the impact of heavy drinking on homicide and suicide mortality in Russia, 1956–2002. Addiction. 2006;101:1719–29. 17156171 10.1111/j.1360-0443.2006.01631.x PMC2596667

[19] Ministério da Saúde. Sistema de Informação sobre Mortalidade. Available in http://www2.datasus.gov.br/DATASUS/index.php?area=040701. Acessed 28 Jan 2022.

[20] Lockemann U, Heinemann A, Wischhusen F, Ewerwahn J, Püschel K. Frequently misinterpreted blood alcohol concentrations in (sudden) natural and unnatural death. Versicherungsmedizin. 1995;47(1):15–7. 7709500

[21] Carlini EA, Galduróz JC, Noto AR, Carlini CM, Oliveira, LG, Nappo SA, et al. II Levantamento domiciliar sobre o uso de drogas psicotrópicas no Brasil: estudo envolvendo as 108 maiores cidades do país - 2005. São Paulo: Páginas & Letras; 2007.

[22] Ponce JC, Andreuccetti G, Jesus MGS, Leyton V, Muñoz DR. Álcool em vítimas de suicídio em São Paulo. Rev Psiq Clín. 2008;35(supl 1):13–16.

[23] Andreuccetti G, Cherpitel CJ, Carvalho HB, Leyton V, Miziara ID, Munoz DR, Reingold AL, Lemos NP. Alcohol in combination with illicit drugs among fatal injuries in Sao Paulo, Brazil: An epidemiological study on the association between acute substance use and injury. Injury. 2018;49(12):2186–92. 30270012 10.1016/j.injury.2018.09.035 PMC6289625

[24] Hanzlick R. Ethanol Concentration in Decomposing Bodies: Another Look, Less Concern. Am J Forensic Med Pathol. 2009;30(1):8. 10.1097/PAF.0b013e3181873814 19237865

[25] Perez J, Beale E, Overholser J, et al. Depression and alcohol use disorders as precursors to death by suicide. Death Stud. 2022;46(3):619–27. 10.1080/07481187.2020.1745954. 32238058 10.1080/07481187.2020.1745954

[26] Bagge CL, Conner KR, Reed L, et al. Alcohol use to facilitate a suicide attempt: An event-based examination. J Stud Alcohol Drugs. 2015;76(3):474–481. 10.15288/jsad.2015.76.474. 10.15288/jsad.2015.76.474 PMC4440305 25978835

[27] Larkin C, Griffin E, Corcoran P, et al. Alcohol involvement in suicide or self-harm. Crisis. 2017;38(6):413–22. 10.1027/0227-5910/a000488. 29183241 10.1027/0227-5910/a000488

[28] Lasota D, Al-Wathinani A, Krajewski P, et al. Alcohol and the Risk of Railway Suicide. Int J Environ Res Public Health. 2020;17:7003. 10.3390/ijerph17197003. 10.3390/ijerph17197003 PMC7578964 32987939

[29] Polimanti R, Peterson RE, Ong JS, et al. Evidence of causal effect of major depression on alcohol dependence: Findings from the psychiatric genomics consortium. Psychol Med. 2019;49(7):1218–26. 10.1017/S0033191719000667. 30929657 10.1017/S0033291719000667 PMC6565601

[30] Cohen GH, Fink DS, Sampson L, et al. Coincident alcohol dependence and depression increase the risk of suicidal ideation among Army National Guard soldiers. Ann Epidemiol. 2017;27(3):157–63. 10.1016/j.annepidem.2016.12.004. 28139369 10.1016/j.annepidem.2016.12.004 PMC5505073

[31] Britton PC, Stephens B, Wu J, et al. Comorbid depression and alcohol use disorders and prospective risk for suicide attempt in the year following inpatient hospitalization. J Affect Disord. 2015;187:151–5. 10.1016/j.jad.2015.08.029. 26339924 10.1016/j.jad.2015.08.029

[32] Sher L, Oquendo MA, Richardson-Vejlgaard R, et al. Effect of acute alcohol use on the lethality of suicide attempts in patients with mood disorders. J Psychiatr Res. 2009;43(10):901–5. 10.1016/j.psychires.2009.01.005. 19246050 10.1016/j.jpsychires.2009.01.005 PMC3767468

[33] Lange S, Jiang H, Kaplan MS, et al. Association Between Acute Alcohol Use and Firearm-Involved Suicide in the United States. JAMA Netw Open. 2023;6(3):e235248. 10.1001/jamanetworkopen.2023,5248. 36988957 10.1001/jamanetworkopen.2023.5248 PMC10061235

[34] Udesen CH, Hviid SS, Becker U, et al. Alcohol-related mortality in 15–24-year-olds in Denmark (2010–2019): a nationwide exploratory study of circumstances and socioeconomic predictors. Lancet Reg Health Eur. 2023;29:100620. 10.1016/lanepe2023.100620. 10.1016/j.lanepe.2023.100620 PMC10070885 37025107

[35] Salles J, Tiret B, Gallini A, et al. Suicide Attempts: How Does the Acute Use of Alcohol Affect Suicide Intent? Suicide Life Threat Behav. 2019;50(1):315–28. 10.1111/sltb.12586. 10.1111/sltb.12586 31532854

[36] Sellers CM, Iriarte ADV, Battalen AW, et al. Alcohol and marijuana use as daily predictors of suicide ideation and attempts among adolescents prior to psychiatric hospitalization. Psychiatry Res. 2019;273:672–7. 10.1016/j.psychres.2019.02.006. 31207851 10.1016/j.psychres.2019.02.006 PMC9262037

[37] Zang X, Wu LT. Suicidal ideation and substance use among adolescents and young adults: a bidirectional relation? Drug Alcohol Depend. 2014;142:63–73. 24969957 10.1016/j.drugalcdep.2014.05.025 PMC4129651

[38] Sher L. Alcohol and Suicide: Neurobiological and Clinical Aspects. Sci World J. 2006;6:700–6. 10.1100/tsw.2006.146. 10.1100/tsw.2006.146 PMC5917128 16799741

